# Active participation and inclusion of children and young people with disabilities in health research in sub-Saharan Africa: a scoping review protocol

**DOI:** 10.1136/bmjopen-2026-120168

**Published:** 2026-08-10

**Authors:** Denis Ndekezi, Andrew Sentoogo Ssemata, Rehema Namaganda, Fiston Gatera Kitema, David Musendo, Joyce Olenja, Janet Seeley, Femke Bannink Mbazzi

**Affiliations:** 1 MRC/UVRI and LSHTM Uganda Research Unit, Entebbe, Central Region, Uganda; 2 International Centre for Evidence in Disability, London School of Hygiene & Tropical Medicine, London, UK; 3 Lifetime Research Group, Kigali, Rwanda; 4 University of Rwanda, Kigali, Rwanda; 5 Department of Public and Global Health, University of Nairobi, Nairobi, Kenya; 6 Department of Global Health and Development, London School of Hygiene & Tropical Medicine, London, UK

**Keywords:** Disabled Persons, Health Services Accessibility, Young Adult, Patient Participation, Community-Based Participatory Research

## Abstract

**Abstract:**

**Introduction:**

Children and young people with disabilities make up a substantial population in sub-Saharan Africa yet their active participation and inclusion in health research remain limited. Although disability-inclusive and participatory approaches are increasingly recognised as essential for ethical and impactful research, evidence on how children and young people with disabilities are actively involved across research stages is inconsistent. This scoping review aims to systematically map and synthesise existing literature on the active participation and inclusion of children and young people with disabilities in health research in sub-Saharan Africa and identify reported interventions, strategies and outcomes that support such involvement.

**Methods and analysis:**

This review will follow the Arksey and O’Malley framework and will be reported according to PRISMA-ScR guidelines. A comprehensive search of MEDLINE, PubMed, Web of Science, Scopus, PsycINFO, Africa-Wide Information and grey literature sources will identify studies published in English from 2000 to 2025. Eligible evidence will include qualitative, quantitative and mixed-methods studies, as well as organisational reports addressing participation of children and youth aged 0–35 years with disability in health research.

All identified records will be imported into Rayyan (Rayyan Systems Inc., Cambridge, MA, USA) for title and abstract screening. Two reviewers will independently screen articles, extract data using a structured charting form and synthesise findings using descriptive and thematic analysis. Any disagreements will be resolved through discussion or consultation with a third reviewer.

**Ethics and dissemination:**

Ethical approval is not required as this review draws solely on publicly available literature. The findings will provide a regional synthesis of disability-inclusive health research involving children and young people in sub-Saharan Africa and will inform the development of equitable, participatory research practices. Results will be disseminated through peer-reviewed publications and accessible formats, including plain-language summaries and youth-friendly presentations.

**STRENGTHS AND LIMITATIONS OF THIS STUDY**

- This review examines the extent, nature and mechanisms of participation and inclusion of children and young people with disabilities in health research in sub-Saharan Africa.
- It is informed by disability-inclusive and participatory principles, enabling critical analysis of power, agency and equity across the research process.
- Inclusion of grey literature alongside peer-reviewed studies will enhance the understanding of context-specific and practice-based approaches.
- The review is limited to English-language and accessible full-text studies; comprehensive database, grey literature and citation searches will be used to minimise bias, and this will be considered in interpretation.
- Variability in definitions of participation, inclusion and disability may limit comparability; these will be systematically extracted and considered during synthesis.

## Introduction

 Globally, an estimated 1.3 billion people, representing approximately 16% of the world’s population, live with some form of disability; an estimated 317 million of these are children and young people.^1 2^ Sub-Saharan Africa has one of the youngest populations globally, with approximately 70% of its population under the age of 30 years.^3^ Although the number of children and young people with disabilities is substantial, reliable estimates remain limited due to inconsistent reporting systems, methodological differences and under-identification of disability.^4–7^

The United Nations Convention on the Rights of Persons with Disabilities (UNCRPD) recognises the right of persons with disabilities to participate fully and effectively in decisions affecting their lives, including research.^8^ This principle is reflected in the widely adopted maxim ‘Nothing About Us Without Us’, which emphasises that research concerning persons with disabilities should be conducted with their active involvement rather than solely about them.^9^ In this review, active participation is defined as the meaningful and equitable involvement of children and young people with disabilities throughout the health research process, including research priority setting, study design, data collection, analysis, interpretation and dissemination such that their voices, priorities and lived experiences influence research decisions and outcomes.^10 11^ Active participation and inclusion of children and youth with disabilities therefore extends beyond recruitment as research participants to include collaboration, shared decision-making and opportunities for leadership across the research cycle.

Promoting active participation and inclusion of children and youth in health research is increasingly recognised as essential for producing ethical, relevant and high-quality evidence. Existing studies demonstrate that involving children and young people with disabilities can improve the relevance, acceptability and quality of research while empowering participants, strengthening trust between researchers and communities, and ensuring that research better reflects lived experiences.^10–13^ However, despite growing international commitments to disability-inclusive research, children and young people with disabilities continue to be under-represented in health research, particularly in leadership and decision-making roles.^11^

Recent reviews have highlighted important advances in disability-inclusive research while identifying persistent gaps. A scoping review of qualitative health research found that children with disabilities were frequently included as participants but were rarely involved in shaping research questions, methods or dissemination.^11^ Similarly, reviews of participatory disability research in low-income and middle-income countries reported that meaningful participation is often constrained by limited capacity strengthening, unequal power relationships and inconsistent implementation of participatory approaches.^14^ Existing reviews have also shown that people with disabilities remain under-represented in clinical research despite increasing policy commitments to equitable inclusion.^15^ Collectively, these reviews demonstrate growing recognition of active participation but provide limited evidence on how it is implemented, supported and sustained in health research involving children and young people with disabilities, particularly within sub-Saharan Africa.

This evidence gap is especially important in sub-Saharan Africa, where children and young people with disabilities continue to experience barriers to healthcare, education and social participation arising from inaccessible environments, socioeconomic inequalities and under-resourced health systems.^16–19^ Although participatory and disability-inclusive research is increasingly promoted across the region, little is known about the extent and nature of active participation, the strategies used to facilitate participation, the outcomes associated with active involvement, and the remaining evidence gaps.

Therefore, this scoping review aims to map and synthesise the existing literature on the active participation of children and young people with disabilities in health research in sub-Saharan Africa. Specifically, the review will examine the extent and nature of active participation, the interventions and strategies used to facilitate participation, the reported outcomes associated with active involvement and the evidence gaps requiring future research. The findings will contribute to a better understanding of disability-inclusive health research practices and help inform future research, policy and practice across the region.

## Methods

This scoping review protocol will be guided by the methodological framework proposed by Arksey and O'Malley^20^ which provides a systematic and rigorous approach to mapping the breadth and nature of evidence on a given topic. The review protocol will be reported in accordance with the Preferred Reporting Items for Systematic Reviews and Meta-Analyses Protocols (PRISMA-P) guidelines,^21^ while the completed review will be reported following the Preferred Reporting Items for Systematic reviews and Meta-Analyses extension for Scoping Reviews (PRISMA-ScR).

The review will be conducted through the following stages: (1) identifying the research question, (2) identifying relevant studies, (3) study selection, (4) data charting and (5) collecting, summarising and reporting the results. The methods adopted for each stage are described below.

### Identifying research questions

This scoping review aims to systematically map and synthesise the existing evidence on the active participation and inclusion of children and young people with disabilities in health research in sub-Saharan Africa. Specifically, the review will examine the extent and the nature of active participation and inclusion, the strategies used to facilitate meaningful involvement, the reported outcomes associated with such engagement, and the remaining evidence gaps to inform future research and practice.

The review will address the following research questions:

1. What is the extent and nature of active participation and inclusion of children and young people with disabilities in health research in sub-Saharan Africa?
2. What interventions or strategies have been used to facilitate active participation and inclusion of children and young people with disabilities in health research in sub-Saharan Africa?
3. What reported or measured outcomes are associated with the active participation and inclusion of children and young people with disabilities in health research in sub-Saharan Africa?
4. What evidence gaps and future research priorities are identified in the literature regarding the active participation and inclusion of children and young people with disabilities in sub-Saharan Africa?

The eligibility criteria were developed to ensure that studies included in the review directly addressed these research questions and are presented in table 1.

**Table 1:** Inclusion and exclusion criteria

Category	Inclusion criteria	Exclusion criteria
Population	Studies involving children and young people aged 0–35 years with any type of disability participating in health research Studies involving caregivers, parents or professionals will be included only where they report on or support the active participation of children and young people with disabilities (younger children or those requiring supported participation)	Studies involving only caregivers, parents, professionals or adults with disabilities (>35 years) where the perspectives or participation of children and young people with disabilities are not reported separately or cannot be identified
Concept	Studies reporting the active participation or inclusion of children and young people with disabilities in one or more stages of the health research processes (priority setting, design, data collection, analysis, interpretation or dissemination) Studies describing interventions or strategies designed to facilitate participation of people with disabilities Studies describing interventions or strategies to facilitate participation or reporting outcomes of participation	Studies where children and young people with disabilities participate only as research subjects without any active role in the research process (involvement in design, conduct or dissemination) Studies focused solely on service participation, clinical care, education or community programmes without involvement in health research
Context	Studies conducted in any country in sub-Saharan Africa	Studies conducted outside sub-Saharan Africa or where data from sub-Saharan Africa cannot be distinguished
Study design	Qualitative, quantitative and mixed-methods studies, participatory research, programme evaluations and relevant grey literature (NGO and government reports) with empirical findings	Opinion papers, editorials, commentaries, conference abstracts, dissertations, theses and publications without accessible full-text or without empirical data.
Language	Studies published in English	Studies published in languages other than English
Publication date	Studies published between January 2000 and 31 December 2025	Studies published before January 2000

NGO, non-governmental organisation.

### Identifying relevant studies (developing the search)

A comprehensive search strategy was developed in collaboration with an academic librarian at the London School of Hygiene & Tropical Medicine (LSHTM) and piloted in the MEDLINE database. The strategy was designed to identify both published and grey literature reporting the active participation and inclusion of children and young people with disabilities in health research in sub-Saharan Africa. The final full search strategy is provided in online supplemental material (S1) and will be adapted for each database to reflect differences in indexing terms and search functionality.

Electronic database searches will be conducted in the Web of Science core collection, MEDLINE, PubMed, PsycINFO, Scopus and Africa-Wide Information. Grey literature will be identified through Google Scholar and target searches of websites of international and regional organisations including WHO, UNICEF and relevant ministries of health in sub-Saharan Africa to identify relevant policies, programme evaluations and technical reports with empirical basis. The search will combine controlled database search terms developed based on the three core concepts of the review: (1) children and young people with disabilities, (2) active participation and inclusion in health research and (3) sub-Saharan Africa. Synonyms within each concept will be combined using the Boolean operator OR and the three concepts will be combined using AND. Search terms will be informed by the International Classification of Functioning, Disability and Health to ensure comprehensive capture of disability-related terminology.^22^

The final search strategy will be adapted for each database and information source to align with indexing practices and platform requirements. After searching, the studies will be screened against the inclusion and exclusion criteria.

Studies published in English language between January 2000 and 31 December 2025 will be eligible for inclusion. This date range is justified by the coming into force of the CRPD in 2008^23^ and the subsequent global and regional push towards inclusive research and disability rights, making literature from the year 2000 onward particularly relevant for capturing evolving practices.^13^ To enhance retrieval, the reference lists of all included studies will be screened for additional relevant articles, and forward citation tracking will be conducted using Google Scholar and PubMed to identify articles that reference key included papers.

### Study selection

All retrieved records will be exported to EndNote 20 (Clarivate, Philadelphia, PA, USA) for deduplication and subsequently imported into Rayyan (Rayyan Systems Inc., Cambridge, MA, USA)^24^ to facilitate title/abstract (level 1) and full-text screening (level 2). Study selection will be undertaken independently by two reviewers using the predefined eligibility criteria. Prior to formal screening, the reviewers will pilot the eligibility criteria on a random sample (10%) of retrieved records to ensure a shared understanding and consistency in their application. Any uncertainties will be discussed, and the eligibility criteria refined where necessary before screening the remaining records.

Following title and abstract screening, full texts of potentially eligible studies will be assessed independently by the same reviewers. Disagreements at either stage will be resolved through discussion, and where consensus cannot be reached, a third reviewer will arbitrate. Reasons for excluding studies at the full-text stage will be documented, and the study selection process will be reported using a PRISMA flow diagram to ensure transparency and reproducibility.

### Charting the data

Data will be extracted using a standardised data-charting form developed by the review team to ensure consistent and systematic capture of relevant information. The form will be based on the review objectives and research questions and piloted independently by two reviewers using a sample of included studies. It will be refined as needed to improve clarity and completeness before full data extraction. Two reviewers will independently chart the data, with discrepancies resolved through discussion or consultation with a third reviewer if required. In line with scoping review guidance, the charting form will remain iterative and may be modified during the review process if additional relevant information is identified. The data extraction form will collect the following information as described in table 2.

**Table 2:** Information to be extracted

Author(s) and **year**	Full list of authors and publication year
Study title	Full title of the study, article, report or document
Journal or source (full reference)	Journal name, volume, issue, pages or full citation for grey literature (report title, organisation, URL)
Aims or research question(s)	Primary and secondary objectives of the study
Participant characteristics	Age range, disability type(s), gender, sample size and setting (school, community)
Recruitment context	Where and how participants were recruited (schools, NGOs, clinics, community)
Sampling method	Sampling strategy (purposive, convenience, snowball and other quantitative methods)
Study design	Type of study (qualitative, quantitative, mixed methods, case study, participatory action research)
Theoretical or conceptual framework	Any guiding framework (UNCRPD, Ubuntu model of disability, social model of disability, participatory research ethics)
Data collection methods	Techniques used (interviews, focus groups, surveys, photovoice, participatory film)
Data analysis methods	How data were analysed (thematic analysis, descriptive statistics, co-analysis with youth)
Stage of research participation	Phase(s) in which children/young people with disabilities were involved: (1) design, (2) data collection, (3) analysis, (4) dissemination and (5) multiple stages
Method of participation	Specific role or mechanism (co-researcher, advisory board member, co-facilitator, peer interviewer, data collection, data management, data analysis, co-authorship, involvement in dissemination activities)
Intervention (if applicable)	Description of any programme, tool or strategy used to facilitate participation (training, adapted materials, support workers)
Reported outcomes of participation	Impacts on research quality, youth empowerment, policy or practice
Barriers to participation	Challenges reported (stigma, communication barriers, lack of support, inaccessible methods)
Facilitators of participation	Enabling factors (training, peer support, inclusive tools, family involvement)
Geographical context	Country and specific setting in sub-Saharan Africa (urban/rural, school type)
Study limitations (as reported)	Author-identified weaknesses or constraints
Comments/additional notes	Any other relevant observations (ethical considerations, use of local languages, translation methods)

UNCRPD, United Nations Convention on the Rights of Persons with Disabilities.

### Collecting, summarising and reporting the results

**Figure 1:**
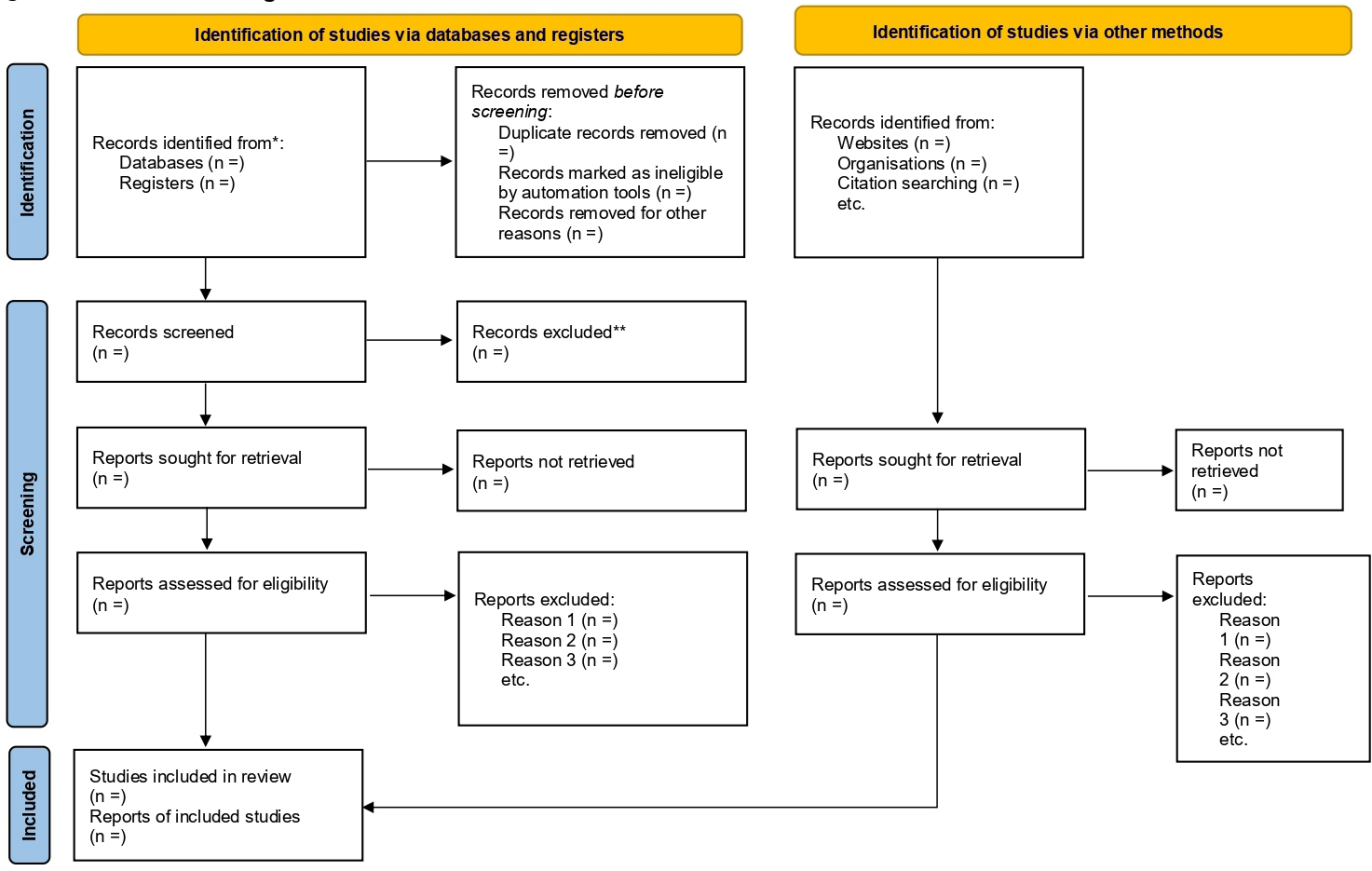
PRISMA 2020 flow diagram. PRISMA 2020 flow diagram illustrating the identification, screening, eligibility assessment and inclusion of studies in the scoping review of the active participation and inclusion of children and young people with disabilities in health research in sub-Saharan Africa. PRISMA, Preferred Reporting Items for Systematic Reviews and Meta-Analyses.

Reporting will follow the PRISMA-ScR.^21^ The study selection process will be presented using a PRISMA flow diagram (figure 1) detailing records identified, screened, assessed for eligibility and included, with reasons for exclusion at the full-text stage.

Study characteristics will be summarised using descriptive statistics and presented in tables and narrative form. Quantitative findings (study and participant characteristics, study designs and stages of participation) will be summarised using frequencies and counts where appropriate. Qualitative findings (experiences, barriers, facilitators and strategies supporting active participation and inclusion) will be synthesised narratively using thematic analysis to identify recurring patterns. Findings will be synthesised by developmental stage (eg, children, adolescents and young adults) to examine how approaches to participation, inclusion and reported outcomes differ across age groups. This will help identify age-specific considerations for designing and implementing disability-inclusive health research.

For mixed-methods studies, quantitative and qualitative findings will be extracted and synthesised separately before being integrated within the overall narrative synthesis. This approach will support a comprehensive understanding of the extent and nature of active participation and inclusion, strategies facilitating meaningful involvement, reported outcomes and gaps in the evidence.

The final synthesis will be organised around the review’s research questions, highlighting the extent and nature of participation, approaches used to facilitate it, reported outcomes and evidence gaps to inform future research, policy and practice.

### Ethics and dissemination

Ethical approval is not required for this scoping review because it synthesises evidence from publicly available literature and does not involve human participants, identifiable personal data or access to confidential information. For queries relating to research governance or research integrity, the The Medical Research Council/Uganda Virus Research Institute and London School of Hygiene & Tropical Medicine Uganda Research Unit (MRC/UVRI and LSHTM) Research Governance Office can be contacted at Regulatory@mrcuganda.org

The review will map and synthesise evidence on the participation and inclusion of children and young people with disabilities in health research across sub-Saharan Africa, focusing on methodological approaches, contextual influences and reported outcomes. This will be the first review to explore this topic within the region, contributing to the advancement of disability-inclusive research practices.

The review’s findings are expected to identify effective strategies for meaningful involvement, highlight evidence gaps and guide future participatory health research. Results will be shared through peer-reviewed publications and accessible formats such as plain-language summaries, infographics and youth-led presentations to reach researchers, policymakers, disability organisations and youth researchers. This inclusive dissemination approach promotes equitable knowledge sharing and supports rights-based research principles.

### Patient and public involvement

None.

## Supplementary material

10.1136/bmjopen-2026-120168 online supplemental file 1
