# Supplementary material for: Active participation and inclusion of children and young people with disabilities in health research in sub-Saharan Africa: a scoping review protocol
Source: BMJ Open. 2026 Aug 10;16(8):e120168. doi: 10.1136/bmjopen-2026-120168 (PMC13475139; doi:10.1136/bmjopen-2026-120168)
Supplement: Supplementary file 1 [file bmjopen-2026-120168.reviewer_comments.pdf]

PEER REVIEW HISTORY

BMJ Open publishes all reviews undertaken for accepted manuscripts. Reviewers are asked to complete a checklist review form and are provided with free text boxes to elaborate on their assessment. These free text comments are reproduced below.

ARTICLE DETAILS

Title (Provisional)

Active participation and inclusion of children and young people with disabilities in health research in sub-Saharan Africa: A Scoping Review protocol

Authors

Ndekezi, Denis; Ssemata, Andrew Sentoogo; Namaganda, Rehema; Kitema, Fiston Gatera; Musendo, David; Olenja, Joyce; Seeley, Janet; Bannink Mbazzi, Femke

---

VERSION 1 - REVIEW

**Reviewer**	**1**
**Name**	**Kumurenzi, Anne**
**Affiliation**	**University of Rwanda College of Medicine and Health Sciences, Physiotherapy**
**Date**	**01-Jul-2026**
**COI**	**None**

Review for Active participation and inclusion of children and young people with disabilities in health research in sub-Saharan Africa: A Scoping Review protocol

General comments:

The protocol presents a comprehensive process that will be followed to conduct a scoping review entitled “Active participation and inclusion of children and young people with disabilities in health research in sub-Saharan Africa: A Scoping Review protocol”, offering valuable insights into the active participation of this population in the sub-Saharan African countries. The research question of the protocol is clearly defined, and the process of the protocol aims to address a gap in the literature on participation and inclusion of children and young people with disabilities in health research in sub-Saharan Africa. However, there are a few areas in the methodology that require revision for clarity, consistency, and scientific rigor as indicated in the attached protocol.

**** **The reviewer provided a marked copy with additional comments. Please contact the publisher for full details.******

VERSION 1 - AUTHOR RESPONSE

Reviewer: 1

General comments:

The protocol presents a comprehensive process that will be followed to conduct a scoping review entitled "Active participation and inclusion of children and young people with disabilities in health research in sub-Saharan Africa: A Scoping Review protocol", offering valuable insights into the active participation of this population in the sub-Saharan African countries. The research question of the protocol is clearly defined, and the process of the protocol aims to address a gap in the literature on participation and inclusion of children and young people with disabilities in health research in sub-Saharan Africa. However, there are a few areas in the methodology that require revision for clarity, consistency, and scientific rigor as indicated in the attached protocol.

Abstract:

Introduction

1. "Search for"

Response: The wording has been corrected for clarity and grammatical accuracy.

Strength and limitation

1. For a protocol, you shouldn't have limitations. How would you go about addressing the limitation

Response: we have retained the "Strengths and limitations of this study" section, in line with the journal's requirements, but revised the anticipated limitations to describe the strategies that will be employed to minimise their impact during the review. Specifically, each limitation is now accompanied by a mitigation strategy, providing greater methodological transparency.

2. Why restrict yourself to English since you have people from French speaking countries?

   Response: We have retained the English language restriction because of the language proficiency of the review team and the resources available for translation. We have acknowledged that this may result in the omission of some relevant studies published in other languages and have included this as an anticipated limitation.

3. The variations in definitions of participation, inclusion, and disability across studies may limit comparability and challenge consistent synthesis of findings. For a protocol, you need to find ways of addressing this"

   Response: We have revised the anticipated limitations to explain how variations in terminology and definitions will be managed during data charting and narrative synthesis. Key concepts will be extracted as reported by the original studies, and

differences in definitions will be explicitly documented and considered during interpretation of the findings.

Introduction.

1. In sub-Saharan Africa, over one billion people reside and approximately 70% are under the age of 30. Re-write this sentence to make it clear.

Response: Thank you. The sentence has been rewritten for improved clarity and readability.

2. However, reliable and comprehensive data remain limited due to inconsistent reporting practices, methodological differences, and persistent discrimination and stigma. data on what?

Response: The sentence has been revised to explicitly state that it refers to data on the prevalence and characteristics of disability among children and young people in sub-Saharan Africa.

Methods

1. Table 1; This table needs major revisions, it's obvious for the readers that what you include in the exclusion column. For example, for the population, you can say studies include children and young people aged 0-35 who have a certain condition or are in a certain state

Response: We have revised Table 1 to improve the clarity and relevance of the eligibility criteria. Rather than listing exclusion criteria that simply mirrored the inclusion criteria, we refined the table to clarify specific eligibility decisions that are likely to arise during study selection. For the population criterion, we now specify that studies involving children and young people aged 0–35 years with disabilities are eligible, including studies where caregivers, parents or professionals report on or support the active participation of children and young people with disabilities where appropriate.

2. Table 1 population. But this is obvious because you mentioned who is going to be included. Among those in the inclusion, who will be excluded

Response: We revised the exclusion criteria to focus on populations that may initially appear eligible but do not meet the review objectives. Specifically, we clarified that studies involving only caregivers, parents, professionals or adults with disabilities (>35 years) will be excluded where the perspectives or active participation of children and young people with disabilities are not reported separately or cannot be distinguished.

3. Population. same comment as above, the study is all about children, we already know this population won't be included. But it would be trick if you have a population of children under 10 based on the countries who need either their parents/caregivers to provide information for them

Response: We have revised the population eligibility criteria to explicitly account for younger children and those who require supported participation. The revised criteria

now state that studies involving caregivers or parents will be eligible where they provide information on behalf of, or support the active participation of, children and young people with disabilities. This revision acknowledges the developmental and contextual needs of younger children while ensuring that the review remains focused on the active participation and inclusion of children and young people with disabilities in health research.

4. Identifying relevant studies, ... was developed in collaboration with the school research librarian... which school are referring to?

Response: We have revised the manuscript to specify that the search strategy was developed in collaboration with an academic librarian at the London School of Hygiene & Tropical Medicine (LSHTM).

5. I will use... I thought it was a team

Response: The manuscript has been revised throughout to consistently use collective language (e.g., "we" and "the review team") where appropriate, accurately reflecting the collaborative nature of the review.

6. You intend to include mixed, how will these be analyzed?

Response: We have expanded the "Collating, Summarising and Reporting the Results" section to clearly describe how mixed-methods studies will be handled. Specifically, quantitative and qualitative findings from mixed-methods studies will be extracted and synthesised separately using appropriate descriptive and narrative approaches before being integrated within the overall narrative synthesis. This approach ensures that evidence from all study designs contributes appropriately to addressing the review questions.

References

This reference is not up to date

Response: We have carefully reviewed and updated the reference list throughout the manuscript. Where appropriate, outdated references have been replaced with more recent and relevant literature, and all references have been checked for accuracy and consistency with the journal's formatting requirements.
