# Supplementary material for: Active participation and inclusion of children and young people with disabilities in health research in sub-Saharan Africa: a scoping review protocol
Source: BMJ Open. 2026 Aug 10;16(8):e120168. doi: 10.1136/bmjopen-2026-120168 (PMC13475139; doi:10.1136/bmjopen-2026-120168)
Supplement: Supplementary file 2 [file bmjopen-16-8-s001.docx]

**Supplementarily material 1 (S1): Search strategy for Active participation and inclusion of children and young people with disabilities in health research in sub-Saharan Africa:**

**Population**: children and young people, children with disabilities, youth with disabilities, young people with disabilities, child, teenagers, juvenile.

**Disability:** disability, impairment, special needs, people with disabilities, disabled persons, physical, sensory, intellectual, developmental disabilities, multiple impairment

**Participation and Inclusion:** active participation, Inclusion, engagement, involvement, empowerment, co-production, co-creation

**Research focus:** health research: health studies, public health research, disability-inclusive research, participatory research, qualitative research, mixed methods research.

**Geographic Scope:** Sub-Saharan Africa, Angola, Benin, Botswana, Burkina Faso, Burundi, Cabo Verde, Cameroon, Central African Republic, Chad, Comoros, Democratic Republic of the Congo, Djibouti, Equatorial Guinea, Eritrea, Eswatini, Ethiopia, Gabon, Gambia, Ghana, Guinea, Guinea-Bissau, Ivory Coast, Kenya, Lesotho, Liberia, Madagascar, Malawi, Mali, Mauritania, Mauritius, Mozambique, Namibia, Niger, Nigeria, Republic of the Congo, Rwanda, Sao Tome and Principe, Senegal, Seychelles, Sierra Leone, Somalia, South Africa, South Sudan, Tanzania, Togo, Uganda, Zambia, and Zimbabwe.

**Web of science core collection**

**Web of science**

("children" OR "child" OR "adolescents" OR "teenagers" OR "youth" OR "young people" OR "juvenile" OR "children with disabilities" OR "youth with disabilities" OR "young people with disabilities" OR "youth and mentor") AND ("disability" OR "impairment" OR "special needs" OR "people with disabilities" OR "disabled persons" OR "physical disability" OR "sensory disability" OR "intellectual disability" OR "developmental disability" OR "multiple impairments") AND ("participation" OR "active participation" OR "inclusion" OR "engagement" OR "involvement" OR "empowerment" OR "co-production" OR "co-creation") AND ("health research" OR "health studies" OR "public health research" OR "disability-inclusive research" OR "inclusive research" OR "participatory research" OR "qualitative research" OR "mixed methods research" OR "Disability Inclusive Youth" OR "Research Training-Mentorship Programme") AND ("Sub-Saharan Africa")

**Search Strategy PubMed**

("Child"[MeSH] OR child*[tiab] OR children[tiab] OR adolescent*[tiab] OR "Adolescent"[MeSH] OR teenager*[tiab] OR youth[tiab] OR "young people"[tiab] OR juvenile*[tiab] OR "children with disabilities"[tiab] OR "youth with disabilities"[tiab] OR "young people with disabilities"[tiab] OR "youth and mentor"[tiab]) AND ("Disabled Persons"[MeSH] OR disability[tiab] OR disabilities[tiab] OR impairment*[tiab] OR "special needs"[tiab] OR "people with disabilities"[tiab] OR "disabled persons"[tiab] OR "physical disability"[tiab] OR "sensory disability"[tiab] OR "intellectual disability"[tiab] OR "developmental disability"[MeSH] OR "multiple impairments"[tiab]) AND (participation[tiab] OR "active participation"[tiab] OR inclusion[tiab] OR engagement[tiab] OR involvement[tiab] OR empowerment[tiab] OR "co-production"[tiab] OR "co-creation"[tiab]) AND ("Health Research"[tiab] OR "Public Health"[MeSH] OR "public health research"[tiab] OR "disability-inclusive research"[tiab] OR "inclusive research"[tiab] OR "participatory research"[tiab] OR "Community-Based Participatory Research"[MeSH] OR "qualitative research"[tiab] OR "mixed methods research"[tiab] OR "Disability Inclusive Youth"[tiab] OR "Research Training-Mentorship Programme"[tiab]) AND ("Africa South of the Sahara"[MeSH] OR "Sub-Saharan Africa"[tiab])

**OVID**

1. (Child OR Adolescent OR Disabled Children OR adolescents OR teenagers OR youth OR young people OR juvenile OR children with disabilities OR youth with disabilities OR young people with disabilities OR youth and mentors OR Mentor).mp.
2. (Disability OR Disabled Persons OR Physical Disability OR Intellectual Disability OR Developmental Disabilities OR Sensory Disorders OR Disabilities OR impairments OR special needs OR people with disabilities OR disabled persons OR multiple impairments or persons with disabilities)
3. (Community Participation OR Participant Participation OR Social Participation OR participation OR inclusion OR inclusive OR engagement OR involvement OR empower OR co-production OR co creation OR inclusive youth or Disability Inclusive)
4. (Health Research OR Public Health OR "health studies OR public health research OR inclusive research OR disability-inclusive research OR participatory research OR mixed methods research OR qualitative research OR Disability Inclusive Youth OR Research Training or Mentorship Programme)
5. (Africa South of the Sahara OR Sub-Saharan Africa OR East Africa OR West Africa OR Central Africa OR Uganda OR Kenya OR Tanzania OR Rwanda OR Burundi OR Ethiopia OR Nigeria OR Ghana).mp.

Subject heading

7            exp Child/

8            exp Adolescent/

9            exp Disabled Children/

10         exp Child, Disabled/

11         exp Mentors/

12         exp Disabled Persons/

13         exp Persons with Disabilities/

14         exp Intellectual Disability/

15         exp Developmental Disabilities/

16         exp Sensory Disorders/

17         exp Hearing Disorders/

18         exp Vision Disorders/

19         exp Social Participation/

20         exp Community Participation/

21         exp Consumer Participation/

22         exp Patient Participation/

23         exp Social Inclusion/

24         exp Social Integration/

25         exp Community-Based Participatory Research/

26         Public Health research/

27         exp Public Health Practice/

28         exp Biomedical Research/

29         exp Qualitative Research/

30         exp Research/ or exp Biomedical Research/ or exp Humans/ or exp Adult/ or exp Mental Disorders/ or exp COVID-19/ or exp Male/ or exp Public Health/ or exp Health Services Research/ or exp Female/ or Health Research.mp.

31         exp Male/ or exp HIV Infections/ or exp Female/ or exp "Surveys and Questionnaires"/ or exp Qualitative Research/ or exp Research Design/ or exp Aged/ or Mixed Methods Research.mp. or exp Adult/ or exp Health Services Research/ or exp Humans/

32         exp Mentorship/

33         exp Capacity Building/

34         exp Education, Professional/

35         exp Sub-Saharan Africa/

36         7 or 8 or 9 or 10 or 11 3612339

37         12 or 13 or 14 or 15 or 16 or 17 or 18

38         19 or 20 or 21 or 22 or 23 or 24 or 25 or 26 or 27 or 28 or 29 or 30 or 31 or 32 or 33 or 34

39         36 and 37 and 38

40         35 and 39

Wide Information (EBSCO)

(TI (child* OR child OR children OR adolescen* OR adolescent* OR teenager* OR youth OR "young people" OR juvenile* OR "children with disabilities" OR "youth with disabilities" OR "young people with disabilities") OR AB(child* OR child OR children OR adolescen* OR adolescent* OR teenager* OR youth OR "young people" OR juvenile* OR "children with disabilities" OR "youth with disabilities" OR "young people with disabilities"))

AND

(TI(disability OR disabilities OR impairment* OR "special needs" OR "people with disabilities" OR "disabled persons" OR "physical disability" OR "sensory disability" OR "intellectual disability" OR "developmental disability" OR "multiple impairments") OR AB(disability OR disabilities OR impairment* OR "special needs" OR "people with disabilities" OR "disabled persons" OR "physical disability" OR "sensory disability" OR "intellectual disability" OR "developmental disability" OR "multiple impairments"))

AND

(TI(participation OR "active participation" OR inclusion OR engagement OR involvement OR empowerment OR "co-production" OR "co-creation") OR AB(participation OR "active participation" OR inclusion OR engagement OR involvement OR empowerment OR "co-production" OR "co-creation" OR "co-design"))

AND

(TI("health research" OR "health studies" OR "public health research" OR "disability-inclusive research" OR "inclusive research" OR "participatory research" OR "qualitative research" OR "mixed methods research") OR AB("health research" OR "health studies" OR "public health research" OR "disability-inclusive research" OR "inclusive research" OR "participatory research" OR "qualitative research" OR "mixed methods research"))

AND

(TI("sub-Saharan Africa" OR Africa OR "East Africa" OR "West Africa" OR "Southern Africa" OR "Central Africa") OR AB("sub-Saharan Africa" OR Africa OR "East Africa" OR "West Africa" OR "Southern Africa" OR "Central Africa"))
